# The Association of Systemic and Mandibular Bone Mineral Density in Postmenopausal Females with Osteoporosis

**DOI:** 10.3390/medicina60081313

**Published:** 2024-08-14

**Authors:** Ioana Duncea, Cecilia Bacali, Smaranda Buduru, Ioana Scrobota, Oana Almășan

**Affiliations:** 1 Prosthetic Dentistry and Dental Materials Department, Iuliu Hațieganu University of Medicine and Pharmacy, 32 Clinicilor Street, 400006 Cluj-Napoca, Romania; 2 Department of Dental Medicine, Faculty of Medicine and Pharmacy, University of Oradea, 410073 Oradea, Romania

**Keywords:** osteoporosis, menopause, estrogen deficit, bone mineral density, postmenopausal bone loss

## Abstract

*Background/Objectives*: Osteoporosis is a common general disease that mostly affects the skeletal system, including the jawbone. There is a link between systemic and mandibular osteoporosis. This study aimed at assessing the association between systemic (lumbar spine L1–L4, femoral neck, total hip) bone mineral density (BMD) and mandible BMD sites in Romanian postmenopausal females. *Methods*: A total of 97 menopausal patients were studied, 62 with osteoporosis and 35 females with no osteoporosis. For each patient, dual-energy X-ray absorptiometry (DXA) assessments of BMD in the mandible, proximal femur, total hip, and lumbar spine (L1–L4) were performed. Mandibular measurements were performed using the distal forearm software, followed by manual analysis after the bone contour was defined in each case. *Results*: Comparing the osteoporosis and control groups, there were significant differences in BMD at each examined location. The mandibular BMD (1.125 ± 0.181506 g/cm^2^) in the osteoporosis group was considerably smaller than in the control group (1.35497 ± 0.244397 g/cm^2^). Correlations between the BMD at different sites were significant: lumbar spine and femoral neck (r = 0.738, *p* < 0.0001), lumbar spine and total hip (r = 0.735, *p* < 0.0001), lumbar spine and mandible (r = 0.506, *p* < 0.0001), femoral neck and total hip (r = 0.891, *p* < 0.0001), femoral neck and mandible (r = 0.482, *p* < 0.0001), and total hip and mandible (r = 0.466, *p* < 0.0001). *Conclusions*: There were correlations between mandible BMD and lumbar spine, femoral neck, and total hip BMD, suggesting that osteoporosis affects mandibular bone density. BMD assessments at common locations may help predict mandibular BMD and the probability of osteoporosis.

## 1. Introduction

Menopause is a natural biological process that marks the end of the menstrual cycle due to lower hormone levels, characterized by a reduction in estrogen and progesterone levels [1,2]. The organism goes through major alterations throughout menopause due to important hormonal changes [3]. A decrease in osteoblast activity causes a decrease in bone density, which is directly impacted by the ovaries’ reduction in estrogen release [4].

Osteoporosis is characterized by bone weakening, which increases the risk of fractures [5]. The etiology of osteoporosis is due to a mismatch between bone loss and bone formation, with an exaggeration of resorption, a reduction in formation, or a combination of both [6]. There is a link between systemic and mandibular osteoporosis [7,8,9]. Mandibular bone loss is a major cause of oral morbidity in postmenopausal females [10,11,12,13].

The two main risk factors for primary osteoporosis are advanced age and insufficient amounts of estrogen production [4].

Osteoporosis prediction is analyzed based on the structure of the mandibular cortical bone, dual-energy X-ray absorptiometry (DXA) being the “gold standard” in BMD evaluation [14]. Lumbar spine (L1–L4) DXA is commonly used for BMD screening [15]. DXA provides the ability to assess BMD with high consistency and reliability [16]. According to the World Health Organization, osteoporosis has been identified according to the description of BMD, considering bone tissue as having a T-score of or <−2.5 SD, osteopenic bone a T-score between −2.5 SD and −1 SD, and normal bone a value of −1 SD or greater [17,18].

Postmenopausal females have an increased likelihood of osteoporotic fractures due to a BMD decline [19]. The sudden reduction in estrogen serum levels after menopause is a contributor to postmenopausal osteoporosis [20]. Postmenopausal women have a high potential of developing osteoporosis, which can accelerate bone resorption [21].

Bone mineral density (BMD) should be evaluated accurately and regularly to reduce the consequences of osteoporosis [22]. BMD is a crucial indicator for osteoporosis diagnosis and treatment planning [23]. Patients with osteoporosis are frequently prescribed bone-modifying medication to lower their risk of skeletal-related complications [24,25].

Osteoporosis affects the size as well as the quality of the jawbone, as shown that postmenopausal women with this condition had smaller jawbone dimensions compared to women with normal BMD [26].

The research in this study was motivated by the possibility that mandibular BMD might influence the response of postmenopausal women to dental treatments.

The research aimed at assessing the correlation between the systemic BMD at different sites, namely L1–L4, femoral neck, and total hip, with that at the mandibular level, in a menopausal female population.

## 2. Materials and Methods

The Ethics Committee of the Faculty of Medicine and Pharmacy “Iuliu Hațieganu” in Cluj-Napoca, Romania, permitted us to conduct the investigation (approval number 428/24.11.2016). The patients provided written consent to participate after the relevant aspects of the investigation were explained to them. Every patient received a research form which included data concerning personal demographic information, results from radiographic and DXA exams, and clinical examinations.

A total of 97 menopausal females who were randomly selected, without associated diseases, and referred for DXA analysis for BMD were studied. The inclusion criteria consisted of menopausal women (cessation of menstruation for more than 12 months) who were referred to the Endocrinology Clinic Cluj-Napoca, Romania. The exclusion criteria were the following: associated general diseases, specific osteoporosis treatment, and other medication.

After the DXA investigations, the participants were divided into two categories: a study group of 62 women with osteoporosis and a control group of 35 patients without osteoporosis.

All patients underwent DXA assessments of their bone mineral density (BMD) in the mandibular bone, proximal femur, total hip, and lumbar spine (L1–L4).

The determination of the bone mineral density (BMD) was carried out by the dual X-ray absorptiometry (DXA) technique, using the DPX-NT equipment, General Electrics, in the endowment of the Endocrinology Clinic in Cluj-Napoca. 

### DXA Measurement and Analysis Protocol

The DXA equipment’s operating principle consists of assessing the mineral density equivalent to the amount of hydroxyapatite per surface unit of the organic bone matrix.

During the study, the calibration of the DPX-NT device was performed at least 3 times each week, using both the device’s phantom and a HOLOGIC phantom (lumbar spine).

The regions of interest analyzed in the study group were the lumbar spine (L1–L4 segment), femoral neck, total hip, and mandible.

Given the absence at a national level of a reference population to which the results obtained from the DXA measurement can be reported, the data provided by NHANES (National Health and Nutrition Examination Survey) III were taken into account in the calculation of the T score [27]. The regions were evaluated using the DPX-NT Bone Densitometer General Electric (General Electric HealthCare) dedicated software package (DPX-NT enCORE software, GE Madison, WI, USA, https://www.gehealthcare.com/) [28]. For the mandible, the distal forearm software was used, with a further evaluation conducted by hand (g/cm^2^) with no T score [27].

The BMD measurement at the spine and hip level was performed with the patient in a reclining position, with both legs raised at approximately 80–90° to the body, knees bent, and resting on a dedicated support. This position provides attenuation of the physiological lumbar lordosis and allows the optimal assessment of the L1–L4 region. The hip assessment was also performed with the patient in a supine position and the lower limbs slightly apart and internally rotated.

In the case of the lumbar spine and hip, the analysis of the evaluated regions was performed automatically, using a special spine and hip software of the DPX-NT equipment.

When evaluating the mandible, the measurements of BMD were performed by two physicians, and the mean value was chosen. The patient was positioned with the cephalic extremity rotated to the left and the mouth wide open. In the case of mandibular DXA, the absence, at present, of a software application dedicated to the evaluation of this region of interest required the use of the distal forearm software in the measurement; the subsequent analysis was performed manually, by defining the outline of the mandibular bone in each case. The obtained amount of bone minerals was related to the measured area, resulting in the mandibular bone density (g/cm^2^). Unlike the spine and hip, the absence of a population-wide reference group did not allow the results to be expressed as a T-score in the mandible.

The SPSS 22.0 statistics software package (Armonk, NY, USA) was chosen for the statistical analysis. Nominal, ordinal, dichotomous, and continuous parameters were assigned to the dataset. To determine if the continuous data distribution was normal, a Kolmogorov–Smirnov analysis was used. To characterize normally distributed parameters, the mean deviation and standard deviation have been utilized. Pearson correlation and Spearman’s rho were used in the study to determine the association between systemic and mandibular bone mineral density (BMD). Pearson correlation was utilized to assess the relationship between BMD at several anatomical sites (L1–L4, femoral neck, total hip) and mandibular BMD in postmenopausal females, including those with and without osteoporosis. Spearman’s rho was used to assess ordinal variables when the normality conditions for Pearson’s correlation were not met and for ordinal data. The *p*-value was fixed at 0.05 as the statistically significant criterion.

## 3. Results

After the BMD evaluation, 62 women were diagnosed with osteoporosis (study group), with a mean age of 62.42 ± 7.852, while 35 patients had no osteoporosis (control group); mean age: 56.80 ± 7.003 (ANOVA test: F = 12.362, *p* < 0.001).

### 3.1. Comparison of Mean BMD at Different Levels between the Two Groups

The characteristics of age, L1–L4 BMD, femoral neck BMD, total hip BMD, and mandible BMD in the osteoporosis (OP) and control (C) group are shown in Table 1.

Levene’s test for equality of variances and *t*-test for equality of means for L1–L4 BMD, femoral neck BMD, total hip BMD, and mandible BMD are shown in Table 2.

For L1–L4 and femoral neck BMD, the Levene test showed significant differences (*p* = 0.003; *p* = 0.033), in the studied lots, and the Student test revealed significance (*p* < 0.0001).

For the total hip and mandible BMD, there were no differences when Levene was applied (*p* = 0.066; respectively *p* = 0.156), but the Student test showed significance in evaluating group C and group OP (*p* < 0.0001).

**Figure 1:**
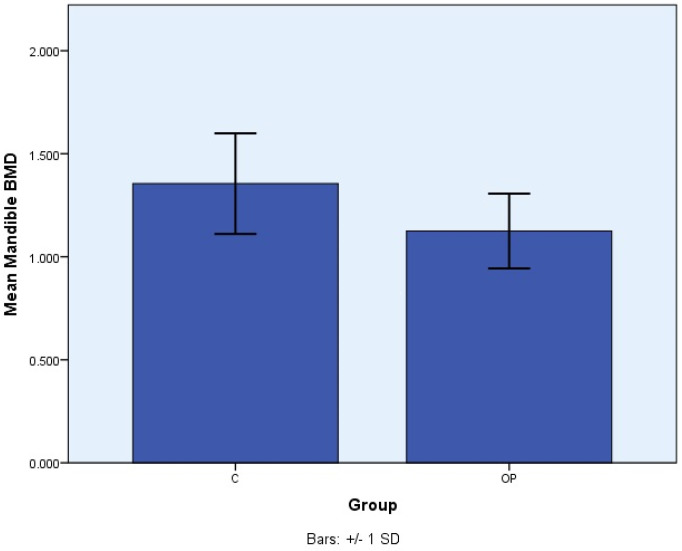
Comparison of mean mandibular BMD in the group of postmenopausal females with no osteoporosis (C) and with osteoporosis (OP). SD—standard deviation.

Figure 1 compares the mandible BMD of postmenopausal females with no osteoporosis (C) and with osteoporosis (OP).

### 3.2. Correlations between BMD at Different Levels

Correlations of the BMD at various levels in the entire patient cohort revealed the subsequent outcomes (Table 3):

There was a moderate to good correlation (r = 0.738, *p* < 0.0001) of the L1–L4 and femoral neck BMD; of the L1–L4 and total hip BMD (r = 0.735, *p* < 0.0001); of the L1–L4 and mandibular BMD (r = 0.506, *p* < 0.0001); and of the femoral neck and total hip BMD (r = 0.891, *p* < 0.0001). There was an acceptable degree of correlation between femoral neck and mandibular BMD (r = 0.482, *p* < 0.0001) and total hip and mandibular BMD (r = 0.466, *p* < 0.0001).

**Figure 2:**
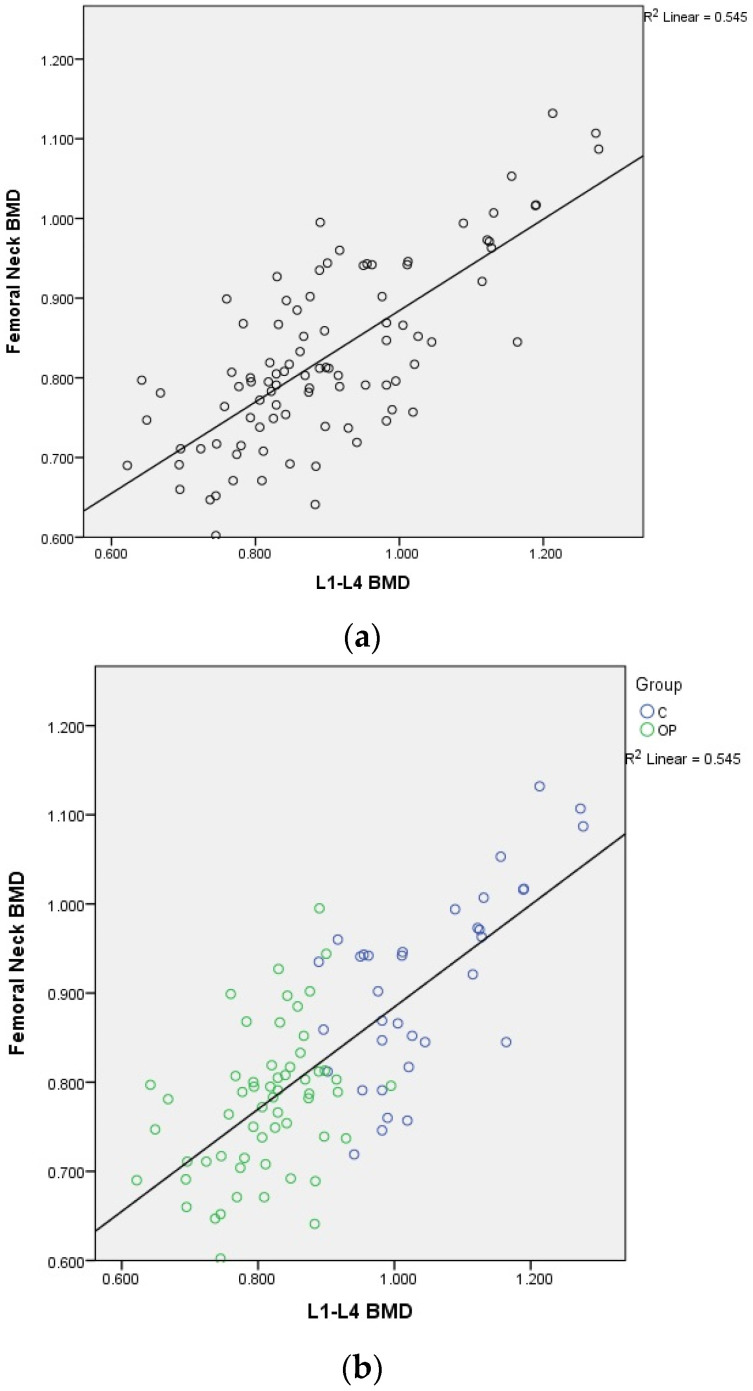
(**a**) Correlation of L1–L4 BMD with femoral neck BMD in postmenopausal women. X-axis: L1–L4 BMD; y-axis: femoral neck BMD (BMD: bone mineral density). (**b**) Correlation of L1–L4 BMD with femoral neck BMD in postmenopausal women. X-axis: L1–L4 BMD; y-axis: femoral neck BMD, grouped by study group (OP) and control group (C) (BMD: bone mineral density).

**Figure 3:**
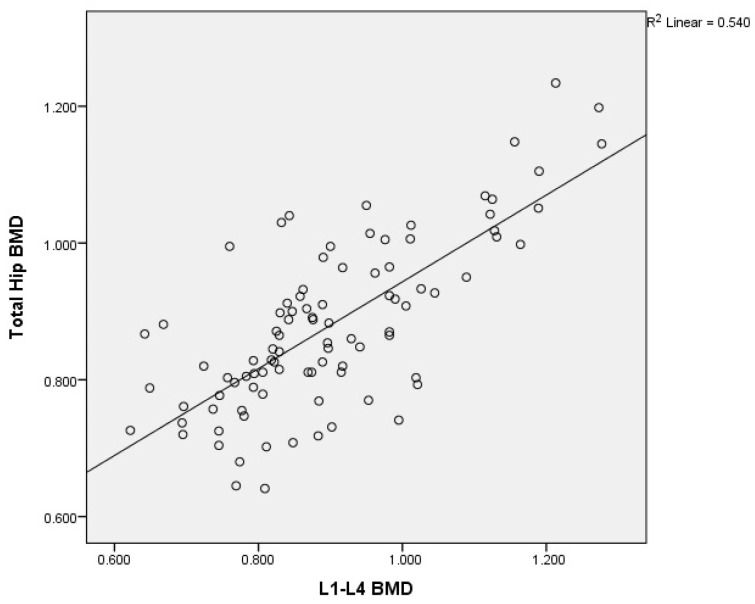
Correlation of L1–L4 BMD with total hip BMD in postmenopausal women. X-axis: L1–L4 BMD; y-axis: total hip BMD (BMD: bone mineral density).

**Figure 4:**
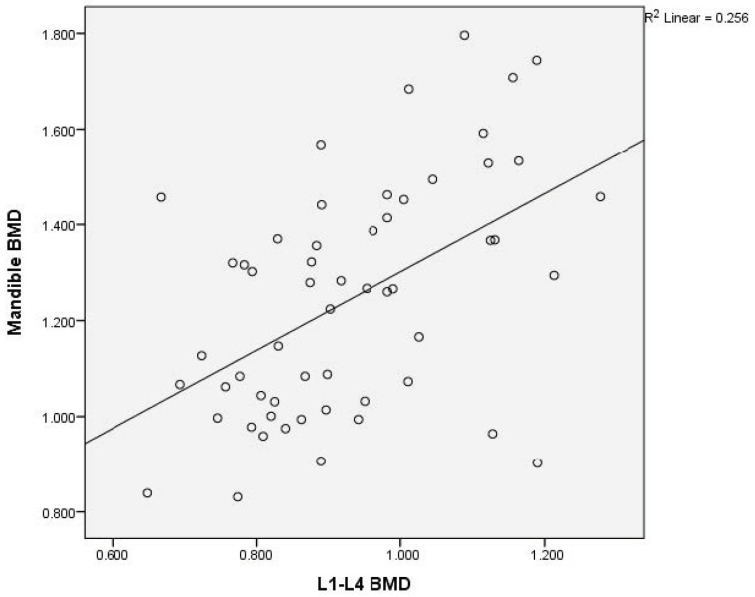
Correlation of L1–L4 BMD with mandibular BMD in postmenopausal women. X-axis: L1–L4 BMD; y-axis: mandibular BMD (BMD: bone mineral density).

**Figure 5:**
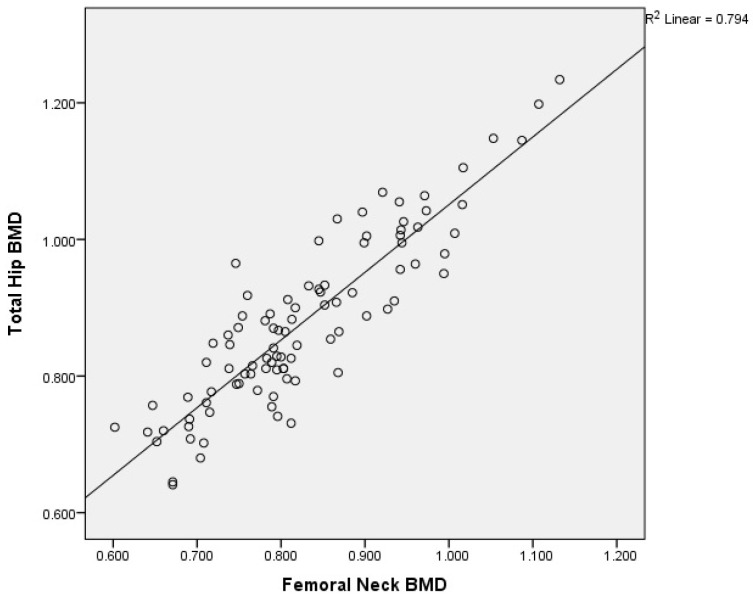
Correlation of femoral neck BMD with total hip BMD in postmenopausal women. X-axis: femoral neck BMD; y-axis: total hip BMD (BMD: bone mineral density).

**Figure 6:**
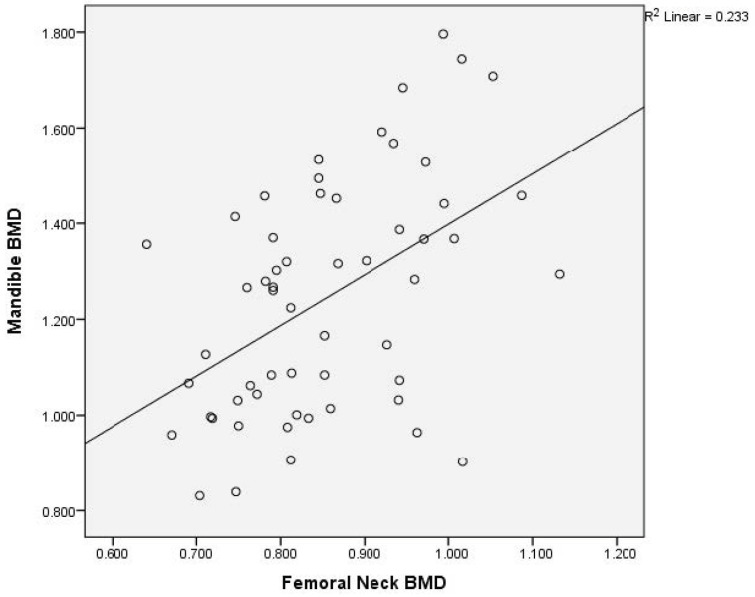
Correlation of femoral neck BMD with mandibular BMD in postmenopausal women. X-axis: femoral neck BMD; y-axis: mandibular BMD (BMD: bone mineral density).

**Figure 7:**
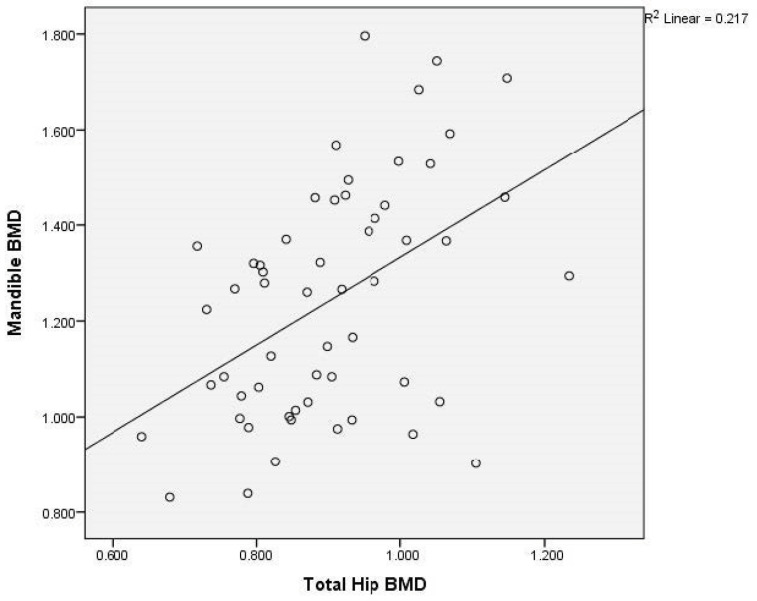
Correlation of total hip BMD with mandibular BMD in postmenopausal women. X-axis: total hip BMD; y-axis: mandibular BMD (BMD: bone mineral density).

Graphical representation of the correlations is illustrated in Figure 2a,b, Figure 3, Figure 4, Figure 5, Figure 6 and Figure 7.

**Figure 8:**
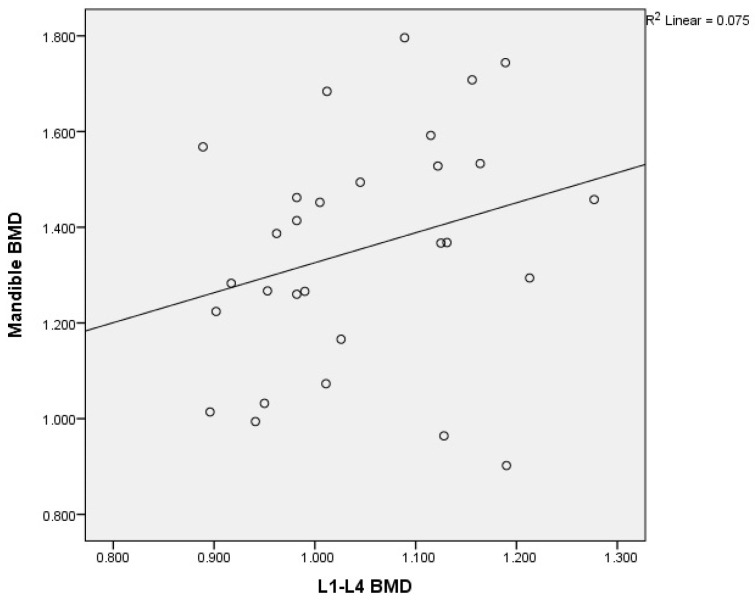
Correlation of L1–L4 BMD with mandibular BMD in postmenopausal women without osteoporosis. X-axis: L1–L4 BMD; y-axis: mandibular BMD (BMD: bone mineral density).

**Figure 9:**
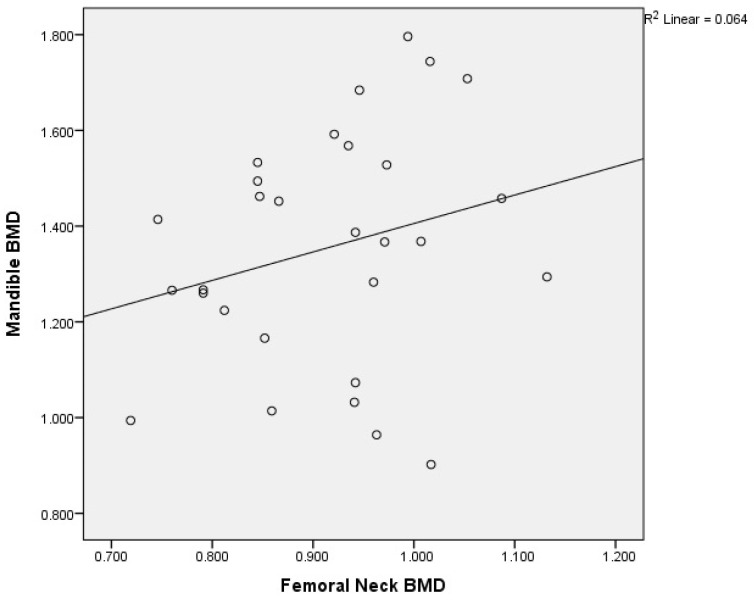
Correlation of femoral neck BMD with mandibular BMD in postmenopausal women without osteoporosis. X-axis: femoral neck BMD; y-axis: mandibular BMD (BMD: bone mineral density).

The correlations of BMD in the patients without osteoporosis (C) revealed the following results (Table 4, Figure 8 and Figure 9):

There was a moderate to good correlation between the L1–L4 and femoral neck BMD (r = 0.685, *p* < 0.0001); between the L1–L4 and total hip BMD (r = 0.755, *p* < 0.0001); and between the femoral neck and total hip BMD (r = 0.843, *p* < 0.0001).

There was an acceptable degree of correlation between the femoral neck and mandibular BMD, (r = 0.252, *p* < 0.093); between the total hip and mandibular BMD (r = 0.203, *p* < 0.146); and between the L1–L4 and mandibular BMD (r = 0.275, *p* < 0.075).

A graphical representation of the correlations of L1–L4 BMD with mandibular BMD in postmenopausal women without osteoporosis is illustrated in Figure 8, and that of the femoral neck BMD with mandibular BMD in postmenopausal women without osteoporosis is shown in Figure 9.

**Figure 10:**
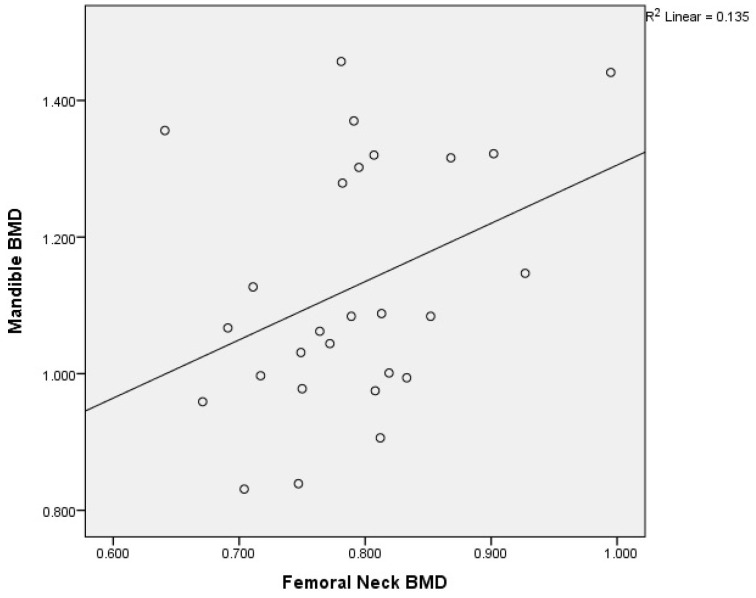
Correlation of femoral neck BMD with mandibular BMD in postmenopausal women with osteoporosis. X-axis: femoral neck BMD; y-axis: mandibular BMD (BMD: bone mineral density).

The correlations of L1–L4 * BMD, femoral neck BMD, total hip BMD, and mandible BMD in females with osteoporosis (OP) revealed the following results (Table 5, Figure 10):

The L1–L4 BMD was not associated with mandibular BMD, but only with femoral neck and total hip BMD. The femoral neck BMD had a correlation with total hip (r = 0.821, *p* < 0.0001) and mandibular BMD (r = 0.367, *p* < 0.030).

## 4. Discussion

This study assessed the association between systemic BMD (L1–L4, femoral neck, total hip) and mandibular BMD levels in postmenopausal females.

In this study, the results showed that the group of postmenopausal women with osteoporosis had a statistically significantly lower mandibular BMD, with a mean BMD of 1.12 g/cm^2^, compared to the group without osteoporosis, with a mean BMD of 1.35 g/cm^2^. This indicates that osteoporosis is not only localized at the levels where DXA is traditionally measured but also at the mandible level.

Age differences were observed in the current study between females with osteoporosis (mean age: 62.42 ± 7.852 years) and the control group (mean age: 56.80 ± 7.003 years). This finding is in agreement with recent research that identified individuals with osteoporosis as having a higher mean age, as the incidence of the disease is higher in elderly patients when the bone structure deteriorates [29,30,31].

Regarding the correlation between BMD values at different levels, in the overall group of postmenopausal women, statistically significant correlations between L1–L4 BMD with femoral neck BMD, total hip BMD, and mandibular BMD, between femoral neck BMD with total hip BMD and mandibular BMD, and between total hip BMD and mandibular BMD were found. In the group of postmenopausal women without osteoporosis, the same correlations as those in the overall group were observed, except for total hip BMD and mandibular BMD. In the group of women with osteoporosis, however, L1–L4 BMD and total hip BMD were no longer associated with mandibular BMD, the rest of the correlations being the same as those in the overall group.

In a study on a possible correlation between osteoporosis and periodontal damage, Von Wowern et al. studied BMD in the mandible and forearm in a group of women with osteoporosis and a control group without osteoporosis, and the results were comparable to our research, with the group with osteoporosis having significantly lower mandibular BMD values than the control group [32].

Osteoporosis, being a generalized disease, is not only limited to the bone tissues traditionally explored by dual X-ray absorptiometry (DXA) but also to the facial bones [33]. In the medical literature in recent decades, the data show that there could be a link between systemic osteoporosis and bone loss in the jaw [34,35,36]. Unfortunately, this hypothesis is difficult to be evaluated, because of the difficulties at the mandibular level, although numerous techniques are used to evaluate osteoporosis at a systemic level [37,38]. Most of the available studies are cross-sectional, and the populations were different in gender, age, the presence of edentulousness, and the prevalence of osteopenia [39].

It has been assumed over time that there is a link between systemic osteoporosis and bone loss in the oral cavity, so radiographs of the alveolar process might prove to be better indicators of systemic osteoporosis than radiographs of other bones [40].

The data are consistent with those in international literature. In Romania, there were no data found on this issue, and thus, a comparison could not be made.

General osteoporosis and decreased density of the bones in the mouth region have been known for years as being associated [41,42,43]. Compared to radiography from different bone structures, radiography of the alveolar region may be a more reliable sign of general osteoporosis [44,45,46,47]. Progressive alveolar bone loss and tooth loss can be associated with osteoporosis [48,49,50]. The loss of alveolar bone with age is similar to that of long bones [51,52,53]. Research on osteoporosis has demonstrated a strong correlation between a decrease in overall bone density and a decrease in jaw density in females with osteoporosis by the use of dual photon absorptiometry [54,55]. Some researchers have suggested that progressive alveolar bone loss with age (leading to tooth loss) is a manifestation of osteoporosis. It appears that the loss of alveolar bone with age is like that of long bones. The use of dual photon absorptiometry to assess osteoporosis has shown that a reduction in total skeletal mass correlates directly with a reduction in jawbone density in women with osteoporosis [41]. In our study, we found a notable correlation between the femoral neck and mandibular BMD.

Humphries et al., investigating toothless mature mandibles, showed that females experienced a considerable decline in bone mineral density as they grew older when compared to men [56,57]. A higher alveolar ridge resorption was found in women compared to men [58,59,60,61].

Erdogan et al. demonstrated an association between decreased mandibular bone density and decreased skeletal bone density, which was also found in this study [62].

Individuals with osteoporosis show a marked decline in bone mineral density in the mandibular body or condyle, including a higher frequency of alveolar crest resorption [26,57,63]. Research on animals has shown that alveolar bone mineral density is susceptible to estrogen deprivation [64]. In the present study, postmenopausal females with osteoporosis also had significantly decreased mandibular BMD. The changes in mandibular BMD observed in this research could be explained, as in the case of systemic BMD, by the action of estrogen at this level as well as the cessation of estrogen production after menopause causing a decrease in BMD, both in skeletal bones and in the mandible.

Payne et al. studied estradiol-sufficient and -deficient females who demonstrated a mean decrease in alveolar mineral content in estradiol deficient females [65]. Though it is not possible to attribute changes in bone structure only to variations in hormone levels, it is necessary to consider additional elements [66,67,68,69]. A reduction in alveolar bony mass might be associated with estrogen insufficiency [50,70].

In contrast to BMD evaluation by DXA, mandible bone mineral density (BMD) is not routinely available, thus establishing that the association between mandible BMD and lumbar spine or hip BMD is of paramount importance to the clinical care of postmenopausal women with dental illness. This study’s novelty is demonstrated by highlighting certain aspects that provide an original and innovative character, such as the use of methods for determining mandibular BMD, that represent relevant and novel diagnostic strategies. These approaches have advanced significantly in recent years. The method represents an objective, non-invasive diagnostic tool to supplement the information provided by clinical and radiologic examinations, resulting in a more accurate diagnosis of mandible osteoporosis. The study’s novelty derives from the absence of prior research conducted in Romania evaluating the relationship between systemic and mandibular BMD in menopausal females by using the DXA method.

Considering the effects of the decrease in mandibular bone density on oral pathology, as well as the fact that the relationships that exist between systemic and mandibular osteoporosis are insufficiently studied and especially quantified, especially in the population of our country, we proposed the approach described in this study.

The oral consequences of osteoporosis are excessive resorption of the residual ridge, tooth loss, pain in the maxillary sinus, chronic destructive periodontal disease, and jaw fractures [6]. Most of the research in the literature on the interrelation between osteoporosis and oral pathology is contradictory, and the populations studied are different, being based on, e.g., age, gender, the presence of edentulism, the prevalence of osteopenia, and other considerations from the point of view of the associated pathology. Also, there is a lack of standardization of methods for evaluating bone mineral density and determining the accuracy and precision of these methods [41].

The analysis of the quality and characteristics of the alveolar bone is highly important when planning prosthetic, surgical (implants), or even orthodontic treatment [71,72,73]. As osteoporosis can promote or aggravate dental problems, the method could help the dentist in the clinical care of postmenopausal women.

Among the limitations of this study are the reduced number of subjects, and other confounding factors that might influence the results like gender, body mass, blood parameters (PTH, Calcium, Vitamin D), and lifestyle. An aspect that might have impacted the sample’s variety was the study population’s geographic limit (a Romanian sample population). The cross-sectional nature of the study implies that it is more challenging to identify the association between systemic and mandibular BMD. Moreover, DXA assessments were used in the study, and DXA analysis has limits in terms of reliability and precision, even though it is frequently utilized for evaluating BMD.

Future research aims at increasing the sample size, standardizing the inclusion criteria, accounting for associated diseases, medication use, and hormone replacement therapy, and considering their impact on systemic and mandibular BMD.

## 5. Conclusions

In postmenopausal women, when comparing the lumbar spine (L1–L4), femoral neck, and total hip BMD with mandibular BMD, a moderate correlation was noticed. The findings indicated a considerable reduction in mandibular BMD in women with osteoporosis when compared to those without the disease. In postmenopausal women with osteoporosis, femoral neck and mandibular BMD were correlated, whereas in postmenopausal women without osteoporosis, mandibular BMD was correlated to femoral neck and L1–L4 BMD.

## Figures and Tables

**Table 1:** Characteristics of age, L1–L4 BMD, femoral neck BMD, total hip BMD, and mandible BMD in the osteoporosis (OP) and control (C) groups.

	Number		Age	L1–L4 * BMD	Femoral Neck BMD	Total Hip BMD	Mandible BMD
Osteoporosis (OP)	62	Mean	62.42	0.80	0.77	0.82	1.12
		SD	7.852	0.07	0.08	0.08	0.18
Control (C)	35	Mean	56.80	1.04	0.91	0.97	1.35
		SD	7.003	0.10	0.10	0.11	0.24

* L1–L4 lumbar spine; BMD: bone mineral density; SD—standard deviation, *p* < 0.001.

**Table 2:** Independent Samples Test for L1–L4 BMD, femoral neck BMD, total hip BMD, and mandible BMD.

		Levene’s Test for Equality of Variances		*t*-Test for Equality of Means					
		F	Sig.	t	Sig. (2-Tailed)	Mean Difference	Std. Error Difference	95% Confidence Interval of the Difference	
								Lower	Upper
L1–L4 * BMD	Equal variances assumed	9.357	0.003	12.640	0.0001	0.235147	0.018604	0.198214	0.272081
	Equal variances not assumed			11.474	0.0001	0.235147	0.020493	0.194046	0.276249
Femoral Neck BMD	Equal variances assumed	4.676	0.033	7.122	0.0001	0.137161	0.019259	0.098911	0.175410
	Equal variances not assumed			6.653	0.0001	0.137161	0.020618	0.095883	0.178439
Total Hip BMD	Equal variances assumed	3.470	0.066	7.024	0.0001	0.151148	0.021518	0.108411	0.193884
	Equal variances not assumed			6.547	0.0001	0.151148	0.023087	0.104920	0.197376
Mandible BMD	Equal variances assumed	2.071	0.156	3.972	0.0001	0.229891	0.057875	0.113859	0.345923
	Equal variances not assumed			4.014	0.0001	0.229891	0.057270	0.114946	0.344836

* L1–L4 lumbar spine; BMD: bone mineral density; STD: standard.

**Table 3:** Correlations of L1–L4 BMD, femoral neck BMD, total hip BMD, and mandible BMD in the entire patient cohort.

		L1–L4 * BMD	Femoral Neck BMD	Total Hip BMD	Mandible BMD
L1–L4 * BMD	Pearson Correlation	1	0.738 **	0.735 **	0.506 **
	Sig. (2-tailed)		0.0001	0.0001	0.0001
Femoral Neck BMD	Pearson Correlation	0.738 **	1	0.891 **	0.482 **
	Sig. (2-tailed)	0.0001		0.0001	0.0001
Total Hip BMD	Pearson Correlation	0.735 **	0.891 **	1	0.466 **
	Sig. (2-tailed)	0.0001	0.0001		0.0001
Mandible BMD	Pearson Correlation	0.506 **	0.482 **	0.466 **	1
	Sig. (2-tailed)	0.0001	0.0001	0.0001	

* L1–L4 lumbar spine; ** correlation is significant at the 0.01 level (2-tailed); BMD: bone mineral density; Sig: significance.

**Table 4:** Correlations of L1–L4 BMD, femoral neck BMD, total hip BMD, and mandible BMD in the control group (C).

		L1–L4 * BMD	Femoral Neck BMD	Total Hip BMD	Mandible BMD
L1–L4 * BMD	Pearson Correlation	1	0.685 **	0.755 **	0.275
	Sig. (1-tailed)		0.0001	0.0001	0.075
Femoral Neck BMD	Pearson Correlation	0.685 **	1	0.843 **	0.252
	Sig. (1-tailed)	0.0001		0.0001	0.093
Total Hip BMD	Pearson Correlation	0.755 **	0.843 **	1	0.203
	Sig. (1-tailed)	0.0001	0.0001		0.146
Mandible BMD	Pearson Correlation	0.275	0.252	0.203	1
	Sig. (1-tailed)	0.075	0.093	0.146	

* L1–L4 lumbar spine (BMD: bone mineral density). ** Correlation is significant at the 0.01 level (1-tailed).

**Table 5:** Correlations of L1–L4 BMD, femoral neck BMD, total hip BMD, and mandible BMD in females with osteoporosis (OP).

		L1–L4 ^ BMD	Femoral Neck BMD	Total Hip BMD	Mandible BMD
L1–L4 ^ BMD	Pearson Correlation	1	0.390 **	0.312 **	0.152
	Sig. (1-tailed)		0.001	0.008	0.225
Femoral Neck BMD	Pearson Correlation	0.390 **	1	0.821 **	0.367 *
	Sig. (1-tailed)	0.001		0.0001	0.030
Total Hip BMD	Pearson Correlation	0.312 **	0.821 **	1	0.315
	Sig. (1-tailed)	0.008	0.0001		0.055
Mandible BMD	Pearson Correlation	0.152	0.367 *	0.315	1
	Sig. (1-tailed)	0.225	0.030	0.055	

^ L1–L4 lumbar spine (BMD: bone mineral density). * Correlation is significant at the 0.05 level (1-tailed). ** Correlation is significant at the 0.01 level (1-tailed).

## Data Availability

The data supporting the findings of the study are available from the corresponding author upon reasonable request.
